# A Retrospective Study of the Seroprevalence of Dengue Virus and Chikungunya Virus Exposures in Nigeria, 2010–2018

**DOI:** 10.3390/pathogens11070762

**Published:** 2022-07-04

**Authors:** Pius S. Ekong, Mabel K. Aworh, Elysse N. Grossi-Soyster, Yiltawe S. Wungak, Nanven A. Maurice, Jonathan Altamirano, Michael J. Ekong, Babasola O. Olugasa, Chika I. Nwosuh, David Shamaki, Bonto Faburay, Desiree A. LaBeaud

**Affiliations:** 1 Central Diagnostic Laboratory, National Veterinary Research Institute, Vom P.M.B 01, Nigeria; pekong@ucdavis.edu (P.S.E.); yiltex2@gmail.com (Y.S.W.); mnanven@hotmail.com (N.A.M.); chikanwosuh@gmail.com (C.I.N.); dshamaki@yahoo.com (D.S.); 2 Veterinary Medicine Teaching and Research Center, School of Veterinary Medicine, University of California Davis, Tulare, CA 93274, USA; 3 Department of Veterinary and Pests Control Services, Federal Ministry of Agriculture & Rural Development, Abuja 900247, Nigeria; mabelaworh@gmail.com; 4 Department of Pediatrics, Division of Infectious Disease, Stanford University School of Medicine, Stanford, CA 94305, USA; elysse@stanford.edu (E.N.G.-S.); altamira@stanford.edu (J.A.); 5 Medical Microbiology and Parasitology Department, University College Hospital, Ibadan P.M.B 3017, Nigeria; mckongmls@gmail.com; 6 Centre for Control and Prevention of Zoonoses, Department of Veterinary Public Health and Preventive Medicine, University of Ibadan, Ibadan P.M.B 3017, Nigeria; b.olugasa@ui.edu.ng; 7 Scientific Liaison Services Section, Foreign Animal Disease Diagnostic Laboratory, National Veterinary Services Laboratories, Animal and Plant Health Inspection Service (APHIS), Plum Island Animal Disease Center, United States Department of Agriculture, New York, NY 11957, USA; bonto.faburay@usda.gov

**Keywords:** arbovirus, dengue, chikungunya, seroprevalence, IgG ELISA, Nigeria

## Abstract

Arboviruses are important public health threats in many regions of the world. Nigeria has experienced outbreaks of arboviruses over the past decades, leading to concerns of widespread endemicity, which are frequently misdiagnosed. This study aimed to determine the seroprevalence of dengue virus (DENV) (a flavivirus) and chikungunya virus (CHIKV) (an alphavirus) infections in three major population centers of Nigeria. A convenience sample of 701 sera was collected from both healthy and febrile participants between August 2010 and March 2018. Sera were tested for prior exposure to CHIKV virus and DENV using indirect IgG ELISA. Results showed that 54.1% (379/701) of participants were seropositive for anti-DENV antibodies, 41.3% (290/701) were seropositive for anti-CHIKV antibodies, and 20.1% (141/701) had previous exposure to both. The seropositivity for prior CHIKV exposure and prior exposure to DENV and CHIKV was significantly associated with age (CHIKV: OR = 2.7 (95% CI: 1.7–4.3); DENV and CHIKV: OR = 2.2 (95% CI: 1.2–4.0) for adults compared to participants under 18 years old). Overall, the high seropositivity across all age groups suggests that arboviral infections are prevalent in Nigeria and indicates that surveillance and further epidemiological studies are required to determine the true burden of these infections and the spectrum of diseases associated with these exposures.

## 1. Introduction

Arboviruses are biologically transmitted by arthropods, such as mosquitoes [1,2,3]. *Aedes aegypti* and *Aedes albopictus* are the mosquito species responsible for transmission of many arboviral species including those under the genera *Alphavirus* and *Flavivirus* [4,5]. Alphaviruses, such as chikungunya virus (CHIKV) and O’nyong nyong virus (ONNV), and flaviviruses, such as dengue virus (DENV), Zika virus (ZIKV), and West Nile virus (WNV), are emerging and re-emerging viruses [6,7,8,9,10]. Dengue fever, caused by DENV, is one the most important mosquito-borne viral diseases with an increasing rise in global incidence. Arboviral infections, such as DENV and CHIKV, can also be transmitted via blood transfusion and possibly vertically from mother to child [11,12]. Although CHIKV has been present mostly in the developing world, chikungunya disease has increasingly been detected in non-endemic countries, indicating threat of further spread to new areas. In Africa, CHIKV and DENV may also be spread via sylvatic cycles in which the virus circulates between forest mosquito vectors and other wild non-human primates [2,13].

When symptomatic, exposure to alphavirus or flavivirus infections often exhibits as a self-limiting, acute febrile illness with a wide range of mild to severe symptoms. These symptoms, many which mimic malaria, may include fever, joint pain, joint swelling, headache, muscular pain, rash, and mild bleeding [14,15]. In Nigeria, malaria parasites transmitted by *Anopheles* mosquitoes have been previously reported to be co-circulating with arboviruses, with many infected individuals presenting co-infection of malaria parasites (*Plasmodium falciparum* and *Plasmodium vivax)* with an arboviral infection [16,17]. Consequently, due to similarities in clinical presentations, it is difficult to distinguish between arboviral infections caused by DENV, CHIKV, or ONNV and malaria [18,19]. This has caused an increased frequency of misdiagnosis and wrong treatments for acute arbovirus infections with lasting sequelae such as encephalitis, hemorrhagic diathesis, and early death [20]. Therefore, investigation of the epidemiology and prevalence of specific arboviral infections in susceptible hosts is critical to understanding disease risk, accurate differential diagnosis and treatment, and the implementation of informed and better-targeted disease control measures.

Nigeria is endemic for many arboviruses, including DENV, CHIKV, WNV, yellow fever virus (YFV), and Rift Valley fever virus (RVFV); however, many outbreaks go undocumented, and the true burden of endemicity remains undetermined due to lack of systematic studies, limited data, insufficient diagnostic capabilities, and misdiagnosis as malaria [14,15,16,17,21,22]. The goal of the present study is to further the understanding of the seroprevalence and risk factors associated with flavivirus and alphavirus infections among individuals from three major population centers in Nigeria. 

## 2. Results

### 2.1. Dengue Virus Exposure

Of the 701 samples tested, 54.1% (*n* = 379, 95% CI: 50.2–57.8%) were positive for the IgG antibody against DENV. The antibody to DENV was detected in 46.2% (*n* = 13, 95% CI: 19.2–74.8%) of newborns within 1–14 days old. Seropositivity varied by location or year of sample collection. The highest seroprevalence, 67.7% (95% CI: 60.3–74.6%), was recorded in Jos, with 62.3% (95% CI: 56.5–67.8%) in Ibadan, and 32.1% (95% CI: 26.0–38.6) in Abuja. In a univariate analysis (Table 1), the odds of anti-DENV antibody seropositivity was about four times higher in Ibadan (OR = 3.49, *p* < 0.001, 95% CI: 2.30–5.12) and Jos (OR = 4.44, *p* < 0.001, 95% CI: 2.85–6.93) than in Abuja. Females had higher risk of anti-DENV antibody seropositivity (OR = 1.41, *p* = 0.02, 95% CI: 1.05–1.91) than males. In addition, persons with symptom of fever at the time of sample collection had approximately two times higher odds of anti-DENV antibody seropositivity (OR = 2.18, *p* < 0.001, 95% CI: 1.42–3.34) than persons without fever. Other significant variables associated with anti-DENV antibody seropositivity included health status (sick individual visiting a health center (OR = 3.81, *p* < 0.001, 95% CI: 2.68–5.42) versus non-sick individuals), previous history of animal exposure (*p* < 0.001), presence of the malaria parasite (*p* < 0.001), and participant occupation (*p* < 0.001) (Table 1). After adjusting for all tested variables in a multivariate analysis, only a previous history of animal exposure and the presence of malaria parasite were significantly associated with anti-DENV antibody seropositivity. Participants diagnosed with malaria evident by the presence of the malaria parasite in the blood were approximately 2.5 times more likely to be seropositive for anti-DENV antibodies (OR = 2.43, *p* = 0.007, 95% CI: 1.27–4.66). Previous history of exposure to domestic and/or food animals was negatively associated with anti-DENV antibody seropositivity (OR = 0.43, *p* = 0.002, 95% CI: 0.25–0.73). 

### 2.2. Chikungunya Virus Exposure

Of the 701 samples tested, 41.3% (*n* = 290 95% CI: 37.6–45.1%) were seropositive for IgG antibody against CHIKV. The antibody to CHIKV was detected in 46.2% (*n* = 13, 95% CI: 19.2–74.8%) of newborns within 1–14 days of age. Seroprevalence for anti-CHIKV antibodies was highest in Abuja (54.0% (95% CI: 47.2–60.6%) in 2011, followed by Jos (37.2%, 95% CI: 30.1–44.8%) in 2017, and Ibadan (34.3%, 95% CI: 28.9–40.0%) in 2018 (Table 2). In a univariate analysis, the odds of anti-CHIKV antibody seropositivity were significantly higher (OR = 2.78, *p* < 0.001, 95% CI: 1.77–4.34) among adults (age group 18–90 years) compared to children (age group 0–17 years). There were lower odds of anti-CHIKV antibody seropositivity in Ibadan/2018 (OR = 0.44, *p* < 0.001, 95% CI: 0.31–0.63) and Jos/2017 (OR = 0.50, *p* < 0.001, 95% CI: 0.33–0.75) compared to Abuja/2011. Participants who were considered sick were less likely to be seropositive for anti-CHIKV antibodies (OR = 0.46, *p* < 0.001, 95% CI: 0.33–0.65) compared to non-sick participants. Additionally, participants positive for malaria parasite were less likely to be seropositive for anti-CHIKV antibodies (OR = 0.48, *p* < 0.001, 95% CI: 0.27–0.86) compared to those negative for the malaria parasite. In a multivariate analysis, only age group and presence of malaria parasite was significantly associated with seropositivity to CHIKV. Seropositivity was significantly higher (OR = 2.15, *p* < 0.001, 95% CI: 1.33–3.45) among adults (age group 18–90 years) compared to children (age group 0–17 years), and presence of the malaria parasite was negatively associated with anti-CHIKV antibodies seropositivity (OR = 0.43, *p* = 0.011, 95% CI: 0.22–0.82).

### 2.3. Prior Dengue Virus L and Chikungunya Virus Exposures

Antibody seropositivity against both DENV and CHIKV was 20.1% (95% CI: 17.2–23.2%), representing 141 of the 701 samples from all study locations. Approximately 7.7% (1/13, 95% CI: 0.2–36.0%) of newborns aged 1–14 days old were seropositive for antibodies against both DENV and CHIKV. Seroprevalence was 20.9% (95% CI: 15.1–27.6%), 21.6% (95% CI: 17.1–26.7%), and 17.4% (95% CI: 12.6–23.0%) in Jos/2017, Ibadan/2018, and Abuja/2011, respectively (Table 3). In a univariate analysis, the odds of being previously exposed to both DENV and CHIKV varied significantly by age groups; adults (age group 18–90 years) had higher odds (OR = 2.26, *p* = 0.007, 95% CI: 1.25–4.08) of seropositivity than children (age group 0–17 years). In a multivariate analysis, only age was significantly associated with seropositivity for antibodies against both DENV and CHIKV. Participants aged 18–90 years old were approximately three times more likely to be seropositivity for antibodies against both DENV and CHIKV (OR = 2.66, *p* = 0.002, 95% CI: 1.44–4.91) compared to younger children aged 0–17 years old.

## 3. Discussion

Overall, the seroprevalence of prior DENV infection was high in the study areas. These rates were consistent with previous studies conducted in Nigeria [15,19] with seroprevalence ranging from 32.1 to 67.7% in our study areas or study year. While we acknowledge the limitations of the broad antibody within the genera *Flavivirus* and *Alphavirus*, the serological assays used in the current study were based on agent-specific diagnostic antigens derived from dengue and chikungunya viruses. Thus, antibodies detected by the assays in this study are considered specific to dengue and chikungunya. Given that study location and sampling period (the year samples were collected) were highly correlated in our study, the observed difference in seroprevalence of prior DENV, CHIKV, or DENV–CHIKV exposures could equally be attributed to both study location and sampling period. A 1977 study by Fagbami et al. [23] describing DENV infections in human sera tested from different geographic locations in Nigeria found a 45% (range: 37–63%) seroprevalence of DENV_2_ among children and adults. As observed in the univariate analysis, the seroprevalence of DENV varied by location/year. Oyero and Ayukekbong [24] in a 2013 study conducted among febrile patients positive or negative for malaria in Ibadan, Nigeria, reported an overall anti-DENV IgG seroprevalence of 73%. Although the high dengue antibody seroprevalence may be attributed to potential cross-reactivity with antibodies due to earlier yellow fever vaccination of the local population or maternal dengue antibodies, in the case of yellow fever vaccination, recent studies demonstrated minimal or absence of cross-reactivity of dengue antibody ELISAs with antibodies raised against yellow fever vaccination [25]. Oyero and Ayukekbong [24] found no gender difference in seroprevalence but a significantly higher IgG seroprevalence among participants with malaria than those without malaria. In the Ibadan/2018 samples collected from febrile participants, we recorded a slightly lower IgG seroprevalence of 62.3% (95% CI: 56.5–67.8%). Conversely, in a 2015–2016 study conducted among febrile patients attending a tertiary hospital in Jos, Nigeria, 27.9% of participants were found to be positive for anti-DENV antibodies with ages 21–30 years mostly affected [26]. In our Jos/2017 study, we recorded a higher seroprevalence of IgG antibodies (67.7% 95% CI: 60.3–74.6%) among febrile patients. In a similar study conducted among febrile patients attending a tertiary hospital in Maiduguri, in northeast Nigeria (a city about 585 km northeast of Jos), from January to May 2015, 37.4% (34/91) of patients were positive for DENV IgM antibodies [27]. A higher seroprevalence of 76.9% (10/13) was recorded among patient aged 1 to 15 years in the Maiduguri study. In Nnewi (a town about 447 km southeast of Ibadan) Nigeria, a 2016–2017 study investigating the seroprevalence of DENV among 0–5 year old children with febrile illness [28] recorded 77.1% (74/96) DENV IgM seropositivity with 43.2% of 4–5 year old, 33.8% of 1–3 year old, 6.8% of 7–12 months, and 16.2% of 0–6 months old seropositive. Conversely, in a study of 91 voluntary non-febrile blood donors aged 18–47 years in Osogbo (a town about 95 km northeast of Ibadan), Nigeria, from January to December 2015, only 2.2% (2/91) of participants were seropositive for DENV IgM antibodies [29]. Otu et al. [30] in a 2017 study reported 6% (24/420) seropositivity for DENV among febrile patients with or without malaria attending health facilities in Cross River State (approximately 724 km southeast of Ibadan), Nigeria. Differences in prevalence by geographic locations and/or sampling year/period could be attributed to variations in climatic conditions, rapid urbanization, expansion of vectors into new regions, and human migration [22]. Our adjusted estimates of DENV risk factors showed significant associations only for presence of the malaria parasite and previous history of animal exposure.

The seroprevalence of prior CHIKV infection in our study was high, ranging from 34% to 54% by study location/year of sample collection. Our estimated prevalence was comparable to the 50.1% (143/285) plaque reduction neutralization test anti-CHIKV antibody prevalence recorded by Baba et al. [16] in febrile, clinically suspected malaria/typhoid patients visiting University of Maiduguri Teaching Hospital, Borno, Nigeria, between July and December 2008. Additionally, they reported a significant difference in the distribution of age in CHIKV-positive patients. In Ilorin, Nigeria, Kolawole et al. [15] recorded 13% (23/176) prevalence of anti-CHIKV IgG antibody in febrile patients; this implies minimal circulation of the virus in the area. In addition, they reported significantly different seroprevalence of CHIKV IgG antibodies among the different age groups. In our study, participants aged 18–90 years were approximately two times more likely to be seropositive for anti-CHIKV IgG antibodies (OR = 2.15) compared to younger (0–17 years) participants. This finding is suggestive of continuous exposure to CHIKV in the study population, with older subjects potentially having more time to be exposed to infected vectors. In addition, the detection of the IgG antibody to CHIKV among 23.0% of subjects aged 0–17 years old is indicative of an ongoing transmission of CHIKV in the study population and/or sampling period. The presence of malaria parasites in participant blood was significantly negatively associated with CHIKV exposure, which suggests a possible lower prevalence of prior malaria-CHIKV exposure in the study population/sampling period.

Prior exposures for both DENV and CHIKV were high in our study cohort (20.1%). This finding is comparable to the estimate reported by Baba et al. [21] from a 2008 study among febrile, clinically suspected malaria/typhoid patients in Borno State, Nigeria. Their study reported 17.8% prevalence of CHIKV–DENV co-infections among study subjects. CHIKV–yellow fever virus, CHIKV–WNV, and DENV–WNV co-infections were also reported. A similar study conducted in 2014 among 60 suspected febrile patients visiting a health center in Simawa, Ogun state, Nigeria [17], found 0.0% CHIKV–DENV co-infection. This study had fewer participants, which may explain the low prevalence of DENV–CHIKV co-infection recorded. However, they reported 24% of participants with presence of malaria and past exposure to CHIKV infection and a 3% malaria with past exposure to DENV (IgM) infection. In our study, the prevalence of malaria–DENV co-infections (65%) among study subjects was two times higher compared to the prevalence of malaria–CHIKV co-infections (31%) in the same population. A systematic review study published in 2018 described the prevalence and distribution of co-infections of malaria, dengue, and chikungunya [31]. The systematic review study reported prevalence as high as 50%, 23%, and 15% for DENV–CHIKV, malaria–DENV, and malaria–CHIKV co-infections, respectively. Their findings indicated a lower percentage of people with malaria–CHIKV co-infections, consistent with our findings.

In our study, participant aged 18–90 years were approximately three times more likely to have been exposed at some point to DENV and CHIKV (OR = 2.66) compared to participants ≤ 17 years old, although a prevalence of 11.1% DENV and CHIKV prior exposures was reported among participants ≤ 17 years old. This finding further suggests more recent virus transmission of both DENV and CHIKV in the study regions. In addition, the detection of the IgG antibody to DENV, CHIKV, and both among newborns could be suggestive of mother–child transmission of the virus in the study population, although we do not have sufficient data to support this theory in our cohort. The incidence of perinatal transmission of DENV and CHIKV has been reported from other studies [32,33,34,35,36,37] with various complications including maternal and infant death [38,39,40]. Similarly, the detection of IgG antibodies in newborn could be suggestive of transplacental antibody transfer from mothers during pregnancy, as found in some studies [41,42]. This finding further suggests exposure in women of childbearing age, which could have harmful effects on their babies [43].

The results reported in our study have limitations due to participant selection bias, limited prior research studies, and time confounding factors. This study included 701 research subjects that were either abattoir workers or febrile patients visiting a hospital/health clinic and thus may not reflect the public in Nigeria, and the responses of the participants may have been influenced by their health status at the time of data collection. Many of the serum samples were collected over several years, and therefore time is a confounding factor in our study. Differences in seroprevalence observed in time and/or study locations may not represent the actual disease trends nor reflect the current disease statuses in the studied population. Furthermore, serological assays performed were limited to the detection of IgG antibodies, which can only be used to describe past exposures. Many previous research studies include detection of IgM antibodies, which indicate a current or recent infection and provide innate immunity to an initial exposure but pose additional challenges in detection. Detection of IgG is also subject to high cross-reactivity within viral genera [44], which hinders the ability to detect specific viral species circulating within our study area. Although we used the CHIKV and DENV antigens to detect the presence of anti-CHIKV and anti-DENV IgG antibodies and controls, which were verified by PRNT, we chose to refer to our results for CHIKV and DENV as past exposure to alphaviruses and flaviviruses, respectively.

The high seropositivity across all age groups from our data suggest arboviral infections are prevalent in Nigeria, with primary concern for flavivirus and alphavirus outbreaks. There is high anti-DENV IgG seroprevalence among younger (0–17 years) and older (18–90 years) participants in our study population, indicating recent exposures with regular viral circulation that should be characterized with further investigation. Additionally, participants 18 years and older were more likely to be seropositive for CHIKV and/or prior exposure to DENV–CHIKV compared to younger participants. We demonstrated young participants with exposure to both viruses, which suggests recent CHIKV and DENV transmission in the area. Further surveillance and additional testing are necessary to identify which viral species are circulating in these regions and to determine the true burden of all infections in Nigeria. 

## 4. Materials and Methods

### 4.1. Study Area and Sample Population

**Figure 1:**
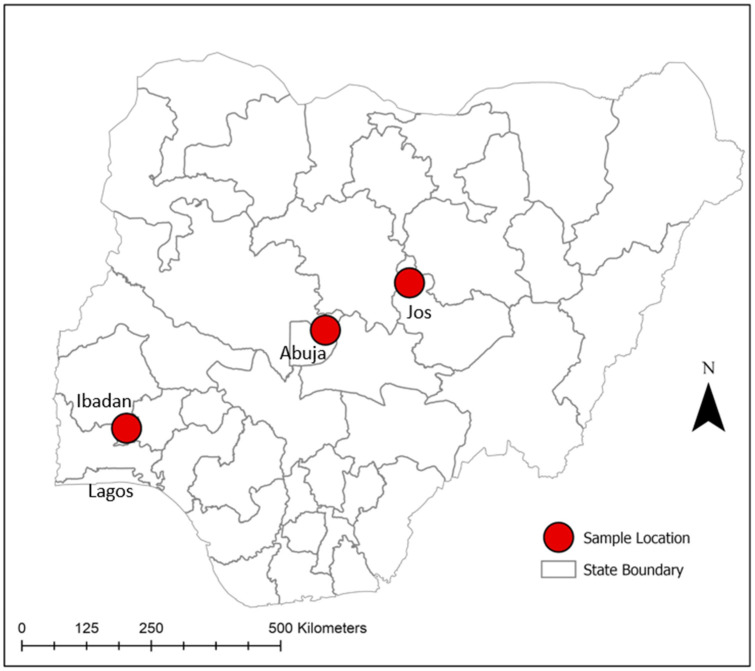
Map of Nigeria showing study locations (Abuja 2011, Jos 2017, Ibadan 2018).

The study was conducted in three major population centers in Nigeria: Ibadan, Abuja, and Jos. Ibadan is in southwest Nigeria, whereas Abuja is located centrally in the Federal Capital Territory, and Jos is within north–central Nigeria (Figure 1). Samples used for this study included banked and freshly collected sera from healthy humans and sick individuals in the study area. For the Abuja cohort, 224 sera samples were originally collected in 2010–2011 in order to investigate the seroprevalence of brucellosis among apparently healthy abattoir workers [45], and they were selected as convenience samples to be tested later for antibodies against flaviviruses and alphaviruses as a part of this study. From Jos, 177 sera samples were collected in 2017 for a study investigating the seroprevalence of brucellosis among suspected febrile individuals attending the Plateau State Specialist Hospital in Jos North local government area (LGA) for treatment, and they were selected to be later tested for antibodies against flaviviruses and alphaviruses as a part of this study. In Ibadan, 300 sera were collected in 2018 from suspected febrile individuals visiting the Saint Mary General Hospital in Ibadan South-East LGA for diagnosis and treatment. For this study, suspected febrile individuals were patients who reported and/or presented with fever or/and other symptoms of febrile diseases such as chills, headache, and joint, muscle, and body pains during the previous 2–4 days and/or at the time of sample collection. Blood samples collected as a part of the Ibadan study were initially tested for the presence of the malaria parasite by microscopy. All sera were transported in a cold chain and stored at −20 °C at the National Veterinary Research Institute (NVRI), Vom, Nigeria.

Additional information, including patient demography such as age, gender, and occupation, history of fever, animal exposure, and malaria diagnosis were recorded at the time of each sample collection. All sera were heat-inactivated at 56 °C for 2 h before being shipped to Stanford University School of Medicine, Department of Pediatrics, Division of Infectious Disease for laboratory analysis. Ethical approval to conduct this study in the three locations was granted by the Plateau State Specialist Hospital Ethical Review Board (PSSH/ADM/ETH.CO/2019/005). Permission was sought from the management of each study site prior to commencement of study. Informed written consent was obtained from participants before being blood sampled and interviewed. Confidentiality of information obtained was assured.

### 4.2. Serological Analysis

Indirect ELISA-detecting anti-CHIKV or anti-DENV immunoglobulin G (IgG) were performed using CHIKV- and DENV_1–4_-specific antigens [19,46], respectively. Antigen preparation and ELISA procedures were performed as described previously [19,46]. Briefly, CHIKV attenuated vaccine strain 181/25 and DENV_1–4_ strains (DENV_1_: Western Pacific 74; DENV2: S16803; DENV_3_: CH53489; and DENV_4_: TVP360) were used to infect Vero cells in Dulbecco’s minimal Eagle medium (DMEM) for viral propagation. After 72 h incubation for CHIKV infections, and 120 h incubation for each individual DENV_1–4_ infection, at 37 °C, supernatant was collected and centrifuged briefly to remove cell debris. Inactivation was performed by treating supernatant-containing viral particles with 5% v/v binary ethyleneimine (BEI) (6.1467 g of BEA (2-bromoethylamine hydrobromide) to 100 mL of 0.2N NaOH) overnight. Inactivated solution was then centrifuged through a 10% sorbitol gradient at 22,000 RPM for 3 h. Pelleted inactivated virus was resuspended in sterile phosphate buffered solution (PBS). Antigen was diluted to a concentration of 25 µg/µL in a 1X PBS coating buffer. A 96-well plate was coated with the diluted antigen by adding 50 ul of antigen-coating buffer solution to each well and incubated overnight at 4 °C. Plates were washed with 1X PBS/0.5% Tween 20/0.01% NaN_3_ wash buffer and blocked using 5% powdered milk in 1X PBS, followed by a 2 h incubation at 37 °C. Plates were washed following the blocking incubation period. Aliquots of 50 μL of serum dilutions (1:100 dilutions in blocking buffer) were added to appropriate wells. Plates were incubated at 4 °C overnight. Subsequently, plates were incubated at 37 °C for an additional hour and washed prior to adding 50 μL of secondary antibody (1:2000 dilution, goat anti-human IgG conjugated to alkaline phosphatase) and incubated at 37 °C for one hour. Plates were then washed, and 100 μL of dissolved alkaline phosphatase tablets (1 mg/1 mL in PnPP buffer) were aliquoted before being incubated at 37 °C for 30 min. The optical density of the plates was read at 405 nm, and cut-off values were calculated to be half of the positive control and twice that of the negative control readings, as described in previous protocols [19]. All controls utilized in these assays were confirmed by the plaque reduction neutralization test (PRNT).

### 4.3. Malaria Microscopy

Thick and thin blood smears were made on clean slides at presentation [47]. Thick films were used for malaria detection. Microscopy was considered negative for malaria if no parasites were observed in the thick smear. Thin smears were used to confirm parasite species and infective stages.

### 4.4. Statistical Analysis

All statistical analyses were performed using STATA statistical software version 15 (StataCorp LLC., TX). Seroprevalence of infection was calculated by determining the percentage of positive samples within each variable category. Differences in seroprevalence across variables were investigated using odds ratios and chi-square tests. Univariate and multivariate logistic regression models were used to test the association between the outcome variables with age, gender, location, occupation, history of animal exposure, health status, presence of fever, and confirmation of malaria parasite in blood. In univariate analysis, variables that were associated with the outcomes at a significance level of 5% (*p* ≤ 0.05) were considered statistically significant. In the multivariate logistic regression, variable selection for analysis was made based on a *p*-value of 0.20 or less (*p* ≤ 0.20) in the univariate regression. In all cases, 95% confidence interval and significance level of 5% were used to determine statistical significance.

## Figures and Tables

**Table 1:** Prevalence and factors associated with dengue virus exposure in humans, Nigeria, 2011–2018.

Variable	*n*/*N*	Seroprevalence % (95% CI)	Unadjusted Odds Ratio (95% CI)	*p*-Value	Adjusted Odds Ratio (95% CI)	*p*-Value
**Age** (continuous)	379/701	54.1 (50.2, 57.8)	1.00 (0.99, 1.01)	0.251	1.00 (0.99, 1.01)	0.757
0–17	78/126	61.9 (52.8, 70.4)	REF			
18–90	301/575	52.3 (48.1, 56.4)	0.67 (0.45, 1.00)	0.052	1.10 (0.71, 1.71)	0.648
**Gender**						
Female	193/329	58.6 (53.1, 64.0)	1.41 (1.05, 1.91)	0.022	0.98 (0.67, 1.44)	0.933
Male	186/372	50.0 (44.8, 55.1)	REF			
**Location, Year**						
Ibadan, 2018	187/300	62.3 (56.5, 67.8)	3.49 (2.38, 5.12)	<0.001	4.26 (0.78, 23.04)	0.092
Jos, 2017	120/177	67.7 (60.3, 74.6)	4.44 (2.85, 6.93)			
Abuja, 2011	72/224	32.1 (26.0, 38.6)	REF			
**Occupation**						
Butcher/Meat seller	45/141	31.9 (24.3, 40.2)	0.30 (0.19, 0.46)	<0.001	2.44 (0.48, 12.61)	0.284
Livestock Trader/Vet	9/27	33.3 (16.5, 53.9)	0.32 (0.12, 0.78)		2.70 (0.45, 16.16)	0.276
Abattoir worker	16/46	34.7 (21.3, 50.2)	0.34 (0.16, 0.68)		3.59 (0.63, 20.30)	0.147
Unknown	120/177	67.7 (60.3, 74.6)	1.34 (0.89, 2.03)		6.51 (1.25, 33.74)	0.026
Others	189/310	60.9 (55.2, 66.4)	REF			
**Animal exposure**						
Unknown	120/177	67.7 (60.3, 74.6)	1.04 (0.62, 1.72)	<0.001		
Yes	168/388	43.2 (38.3, 48.3)	0.37 (0.24, 0.57)		0.43 (0.25, 0.73)	0.002
No	91/136	66.9 (58.3, 74.7)	REF			
**Health status**						
Sick individual	307/477	64.3 (59.8, 68.6)	3.81 (2.68, 5.42)	<0.001		
Non-sick individual	72/224	32.1 (26.0, 38.6)	REF			
**Presence of fever**						
Yes	339/595	56.9 (52.8, 60.9)	2.18 (1.42, 3.34)	<0.001	0.60 (0.33, 1.10)	0.100
No	40/106	37.7 (28.5, 47.6)	REF			
**Malaria parasite**						
Unknown	192/401	47.8 (42.8, 52.8)	0.78 (0.44, 1.38)	<0.001		
Yes	153/237	64.5 (58.1, 70.6)	1.55 (0.84, 2.82)		2.43 (1.27, 4.66)	0.007
No	34/63	53.9 (40.9, 66.6)	REF			

*n* = number positive for anti-dengue virus IgG; *N* = total samples examined; CI = confidence interval.

**Table 2:** Prevalence and factors associated with chikungunya virus exposure in humans, Nigeria, 2011–2018.

Variable	*n*/*N*	Seroprevalence % (95% CI)	Unadjusted Odds Ratio (95% CI)	*p*-Value	Adjusted Odds Ratio (95% CI)	*p*-Value
**Age** (continuous)	290/701	41.3 (37.6, 45.1)	1.01 (1.00, 1.02)	<0.001	1.01 (1.01, 1.02)	<0.001
0–17	29/126	23.0 (15.9, 31.3)	REF			
18–90	261/575	45.3 (41.2, 49.5)	2.78 (1.77, 4.34)	<0.001	2.15 (1.33, 3.45)	0.002
**Gender**						
Female	132/329	40.1 (34.7, 45.6)	0.90 (0.67, 1.22)	0.528		
Male	158/372	42.4 (37.3, 47.6)	REF			
**Location, Year**						
Ibadan, 2018	103/300	34.3 (28.9, 40.0)	0.44 (0.31, 0.63)	<0.001	0.67 (0.16, 2.72)	0.577
Jos, 2017	66/177	37.2 (30.1, 44.8)	0.50 (0.33, 0.75)			
Abuja, 2011	121/224	54.0 (47.2, 60.6)	REF			
**Occupation**						
Butcher/Meat seller	70/141	49.6 (41.1, 58.1)	1.81 (1.21, 2.72)	<0.001	0.63 (0.16, 2.39)	0.500
Livestock Trader/Vet	13/27	48.1 (28.6, 68.0)	1.71 (0.77, 3.77)		0.57 (0.12, 2.57)	0.472
Abattoir worker	32/46	69.5 (54.2, 82.2)	4.21 (2.15, 8.23)		1.66 (0.39, 7.01)	0.491
Unknown	66/177	37.2 (30.1, 44.8)	1.09 (0.74, 1.60)		0.37 (0.09, 1.47)	0.162
Others	189/310	60.9 (55.2, 66.4)	REF			
**Animal exposure**						
Unknown	66/177	37.2 (30.1, 44.8)	0.96 (0.60, 1.52)	0.205	-	-
Yes	172/388	44.3 (39.3, 49.4)	1.28 (0.86, 1.91)		1.17 (0.69, 1.97)	0.550
No	52/136	38.2 (30.0, 46.9)	REF			
**Health status**						
Sick individual	169/477	35.4 (31.1, 39.9)	0.46 (0.33, 0.65)	< 0.001		
Non-sick individual	121/224	54.0 (47.2, 60.6)	REF			
**Presence of fever**						
Yes	237/595	39.8 (35.8, 43.8)	0.66 (0.43, 1.00)	0.051	1.03 (0.59, 1.80)	0.899
No	53/106	50.0 (40.1, 59.8)	REF			
**Malaria parasite**						
Unknown	187/401	46.6 (41.6, 51.6)	0.96 (0.56, 1.63)	<0.001		
Yes	73/237	30.8 (24.9, 37.1)	0.48 (0.27, 0.86)		0.43 (0.22, 0.82)	0.011
No	30/63	47.6 (34.8, 60.5)	REF			

*n* = number positive for anti-*chikungunya virus* IgG; *N* = total samples examined; CI = confidence interval.

**Table 3:** Prevalence and factors associated with multiple prior dengue virus and chikungunya exposure in humans, Nigeria, 2011–2018.

Variable	*n*/*N*	Seroprevalence % (95% CI)	Unadjusted Odds Ratio (95% CI)	*p*-Value	Adjusted Odds Ratio (95% CI)	*p*-Value
**Age** (continuous)	141/701	20.1 (17.2, 23.2)	1.01 (1.00, 1.02)	<0.001	1.01 (1.01, 1.02)	<0.001
0–17	14/126	11.1 (6.21, 17.9)	REF			
18–90	127/575	22.0 (18.7, 25.7)	2.26 (1.25, 4.08)	0.006	2.66 (1.44, 4.91)	0.002
**Gender**						
Female	68/329	20.6 (16.4, 25.4)	1.06 (0.73, 1.54)	0.731		
Male	73/372	19.6 (15.7, 24.0)	REF			
**Location, Year**						
Ibadan, 2018	65/300	21.6 (17.1, 26.7)	1.31 (0.84, 2.03)	0.464		
Jos, 2017	37/177	20.9 (15.1, 27.6)	1.25 (0.75, 2.06)			
Abuja, 2011	39/224	17.4 (12.6, 23.0)	REF			
**Occupation**						
Butcher/Meat seller	21/141	14.8 (9.46, 21.8)	0.64 (0.37, 1.10)	0.285		
Livestock Trader/Vet	4/27	14.8 (4.18, 33.7)	0.64 (0.21, 1.92)			
Abattoir worker	13/46	28.2 (15.9, 43.3)	1.45 (0.72, 2.92)			
Unknown	37/177	20.9 (15.1, 27.6)	0.97 (0.62, 1.53)			
Others	66/310	21.2 (16.8, 26.2)	REF			
**Animal exposure**						
Unknown	37/177	20.9 (15.1, 27.6)	0.76 (0.44, 1.29)	0.134	0.66 (0.38, 1.14)	0.179
Yes	69/388	17.7 (14.1, 21.9)	0.62 (0.39, 0.99)		0.69 (0.41, 1.18)	0.141
No	35/136	25.7 (18.6, 33.9)	REF			
**Health status**						
Sick individual	102/477	21.3 (17.7, 25.3)	1.29 (0.85, 1.94)	0.222	1.46 (0.87, 2.44)	0.148
Non-sick individual	39/224	17.4 (12.6, 23.0)	REF			
**Presence of fever**						
Yes	123/595	20.6 (17.4, 24.1)	1.27 (0.73, 2.19)	0.383		
No	18/106	16.9 (10.3, 25.5)	REF			
**Malaria parasite**						
Unknown	76/401	18.9 (15.2, 23.1)	0.74 (0.39, 1.40)	0.603		
Yes	50/237	21.0 (16.0, 26.8)	0.85 (0.44, 1.65)			
No	15/63	23.8 (13.9, 36.2)	REF			

*n* = number positive for dengue virus and chikungunya IgG; *N* = total samples examined; CI = confidence interval.

## Data Availability

Not applicable.
